# Response to: Myocardial fibrosis and arrhythmogenesis in elite athletes

**DOI:** 10.1002/clc.23225

**Published:** 2019-07-04

**Authors:** Cesare Cuspidi, Marijana Tadic, Carla Sala, Elisa Gherbesi, Guido Grassi, Giuseppe Mancia

**Affiliations:** ^1^ Department of Medicine and Surgery University of Milano‐Bicocca Milano Italy; ^2^ Istituto Auxologico Italiano Milano Italy; ^3^ Department of Cardiology Charité‐University‐Medicine Campus Virchow Klinikum Berlin Germany; ^4^ Department of Clinical Sciences and Community Health University of Milano and Fondazione Ospedale Maggiore IRCCS Policlinico di Milano Milano Italy; ^5^ IRCCS Multimedica, Sesto San Giovanni Milan Italy

To the Editor,

1

We would like to thank Dr. Ahmad et al^1^ on their interest for our recently published meta‐analysis.^2^ There are different opinions regarding the importance of myocardial fibrosis in elite athletes. Some authors found significant myocardial fibrosis in this group,^3^ whereas other authors did not reveal any specific structural changes.^4^ As the Authors mentioned, fibrosis was found mainly in the left and right ventricle, interventricular septum, papillary muscles, and bundle branches.^3^ Fibrosis was predominantly located in the septum and more specifically in the insertion points between left and right ventricle. This is unspecific finding because fibrosis in this portion of myocardium represents the common finding in general population and has not been related with adverse outcome (including arrhythmias). Even the authors who reported myocardial fibrotic changes in athletes claimed that this fibrosis was unspecific.^3^ The question that rises is why these fibrotic changes, if detected at the first place, would be associated with arrhythmias in the elite athletes. Additionally, van de Schoor et al showed patchy or focal fibrosis of the ventricles and not atria,^3^ which was the topic of our article. One could hypothesize that atrial fibrosis is present in the elite athletes, but this would be challenging to confirm even with cardiac magnetic resonance. Namely, patchy or focal late gadolinium enhancement (LGE) in the left atrial wall—the main indicator of myocardial fibrosis, is not easy to detect because of small atrial wall thickness. In patients with atrial fibrillation, LGE of the left atrium is transmural and therefore not difficult to diagnose. However, subtle LGE in athletes is not easily detectable. Furthermore, there are important issues, such as low availability, unfavorable cost‐effectiveness ratio of performing cardiac magnetic resonance in asymptomatic athletes and problem with claustrophobia, which is present in a significant number of subjects undergoing magnetic resonance. On the other hand, echocardiographic examination is fast, affordable, and widely available imaging tool that can be used in the screening of elite athletes worldwide. Left atrial strain analysis nowadays could be performed in most of echocardiographic laboratories, which is crucial for the wide usage of every screening method. More importantly, left atrial strain indirectly indicates left atrial fibrosis^5^ and could be even used for the detection of atrial fibrosis during the screening of athletes instead of comprehensive, long, and expensive examination by cardiac magnetic resonance.

The authors suggested that assessment of fibrosis should be added in the score to predict arrhythmias in elite athletes. However, the predictive importance of fibrosis in athletes on arrhythmias occurrence has to be more detailed examined and we believe that it is too early to include this parameter into the screening of elite athletes. It is quite possible that establishing of this predictive score requires several steps. We believe that comprehensive echocardiographic exam that includes evaluation of left atrial longitudinal strain should be the first step. Considering the fact that echocardiographic examination is not obligatory for elite athletes in the most countries, we strongly believe that this step would significantly decrease cardiovascular morbidity and mortality in elite athletes. Electrocardiogram and functional capacity evaluation cannot be the only screening tests among elite athletes, who are exposed to large physical effort every day. Afterwards, we could consider the evaluation of myocardial fibrosis including atrial fibrosis as the next step of cardiologic evaluation in this specific population.

## CONFLICT OF INTEREST

The authors declare no potential conflict of interests.
